# MiR‐103 inhibiting cardiac hypertrophy through inactivation of myocardial cell autophagy via targeting TRPV3 channel in rat hearts

**DOI:** 10.1111/jcmm.14095

**Published:** 2019-01-03

**Authors:** Hanping Qi, Jing Ren, Mingyao E, Qianhui Zhang, Yonggang Cao, Lina Ba, Chao Song, Pilong Shi, Bowen Fu, Hongli Sun

**Affiliations:** ^1^ Department of Pharmacology Harbin Medical University‐Daqing Daqing China

**Keywords:** autophagy, cardiac hypertrophy, miR‐103, TRPV3

## Abstract

Cardiac hypertrophy is a common pathological change frequently accompanied by chronic hypertension and myocardial infarction. Nevertheless, the pathophysiological mechanisms of cardiac hypertrophy have never been elucidated. Recent studies indicated that miR‐103 expression was significantly decreased in heart failure patients. However, less is known about the role of miR‐103 in cardiac hypertrophy. The present study was designed to investigate the relationship between miR‐103 and the mechanism of pressure overload‐induced cardiac hypertrophy. TRPV3 protein, cardiac hypertrophy marker proteins (BNP and β‐MHC) and autophagy associated proteins (Beclin‐1 and LC3‐II) were up‐regulated, as well as, miR‐103 expression and autophagy associated proteins (p62) were down‐regulated in cardiac hypertrophy models in vivo and in vitro respectively. Further results indicated that silencing TRPV3 or forcing overexpression of miR‐103 could dramatically inhibit cell surface area, relative fluorescence intensity of Ca^2+^ signal and the expressions of BNP, β‐MHC, Beclin‐1 and LC3‐II, but promote p62 expression. Moreover, TRPV3 protein was decreased in neonatal rat ventricular myocyte transfected with miR‐103, but increased by AMO‐103. Co‐transfection of the miR‐103 with the luciferase reporter vector into HEK293 cells caused a sharp decrease in luciferase activity compared with transfection of the luciferase vector alone. The miR‐103‐induced depression of luciferase activity was rescued by an AMO‐103. These findings suggested that TRPV3 was a direct target of miR‐103. In conclusion, miR‐103 could attenuate cardiomyocyte hypertrophy partly by reducing cardiac autophagy activity through the targeted inhibition of TRPV3 signalling in the pressure‐overloaded rat hearts.

## 1 INTRODUCTION

Cardiac hypertrophy is an adaptive response to the increased blood pressure and afterload, and is also a common pathophysiological process in many cardiovascular diseases such as hypertension, myocardial infarction, valvular heart disease, cardiomyopathy and so on.^1^, ^2^ Despite, the hypertrophic response is a compensatory mechanism that augments cardiac output, sustained hypertrophy can lead to dilated cardiomyopathy, heart failure and sudden cardiac death. In the western country, approximately half a million individuals are diagnosed with heart failure each year, with a mortality rate approaching 50%. There have been major advances in the identification of genes and signalling pathways involved in this disease process, but the overall complexity of hypertrophic remodelling suggests that additional regulatory mechanisms remain to be identified. Therefore, identifying new molecular mechanisms mediating cardiac hypertrophy and providing new targets for clinical treatment need to be addressed.

MicroRNAs (miRNAs), as the endogenous, non‐coding and single‐stranded RNAs of approximate 22 nucleotides, can directly regulate more than 30% of the genes in a cell,^3^ which are major regulators of cell differentiation, growth, proliferation and apoptosis.^4^, ^5^, ^6^ Similarly, many miRNAs are now also known to participate in regulating cardiac hypertrophy, such as miR‐133, miR‐195, miR‐1^7^, ^8^, ^9^ and so on. MiR‐103 is a member of the miR‐103/107 family located on human chromosome 5.^10^ It is widely distributed in 13 kinds of human normal tissues (liver, placenta, brain, heart, stomach, lung, bladder, prostate, colon, thymus, ovaries, fat and uterus), and five kinds of carcinoma.^11^ Recent studies have also shown that miR‐103 expression was significantly lowered in heart failure patients than healthy volunteers.^12^ However, the role of miR‐103 in protecting against cardiac hypertrophy has never been elucidated.

We used targetscan software to predict the downstream of miR‐103, and found that TRPV3 was the underlying target of miR‐103. TRPV3, a non‐selective cation channel, belongs to the Ca^2+^‐permeant TRP channel family, and which functions in the formation of the skin barrier, hair growth, wound healing and temperature sensation.^13^, ^14^, ^15^, ^16^ TRPV3 can be activated by warm temperatures above 33°C and natural herbs such as carvacrol and inhibited by ruthenium red. In recent years, many studies have expanded the functions of TRP channels, and confirmed that they participated in the regulation of various diseases such as anxiety, asthma, obesity and metabolic disorders.^17^ In particular, TRP channels are also involved in the regulation of cardiovascular disease. TRPV4 is an important regulator of intracellular Ca^2+^ concentration in cardiac fibroblasts.^18^ It has also been reported that TRPV1 was up‐regulated in pathological myocardial hypertrophy.^19^ According to our previous study, TRPV3 activation exacerbated cardiac fibrosis by promoting cardiac fibroblast proliferation through TGF‐β_1_/CDK2/cyclin E pathway in the pressure‐overloaded rat hearts.^20^ Interestingly, the overall sequence of TRPV3 has 40% homology to TRPV1.^21^ Therefore, we speculate whether TRPV3 is also involved in the occurrence of cardiac hypertrophy.

Autophagy is a conserved, tightly regulated intracellular catabolic process, in which mammalian cells degrade and recycle the damaged and dysfunctional macromolecules and organelles. Recent findings suggest that autophagy exploits a variety of physiological functions to maintain the balance of cardiac homeostasis.^22^ For example, a certain extent of autophagy is crucial for proper heart function, whereas exaggerated autophagic activity may foster many cardiovascular diseases such as cardiac hypertrophy, cardiac ischaemia/reperfusion injury, heart failure and so on.^23^, ^24^, ^25^ In tumour cells, intracellular Ca^2+^, an important regulatory factor for autophagy, induced autophagy by death‐associated protein kinase (DAPK).^26^ TRPV3, as a non‐selective cationic channel, can mediate the extracellular Ca^2+^ flow and increase [Ca^2+^]_i_. Hence, we conjectured that TRPV3 channel might participate in cardiac hypertrophy by regulating the autophagy pathway.

The main purpose of the present study was to confirm the following problems: Are miR‐103, TRPV3 and autophagy related to the occurrence of cardiac hypertrophy? Do these three have regulatory effects on cardiac hypertrophy? Are there a regulatory relationship between these three and miR‐103/TRPV3/autophagy regulatory network? The resolution of the above questions would elucidate a novel mechanism of miR‐103/TRPV3/autophagy in cardiac hypertrophy, and provide a new idea for the therapy of pathological cardiac hypertrophy.

## 2 MATERIALS AND METHODS

### 2.1 Pressure overload‐induced cardiac hypertrophy

All experimental procedures conformed to the Guide for the Care and Use of Laboratory Animals published by the US NIH (publication, 8th Edition, 2011). This study, including any relevant details, was approved by the Experimental Animal Ethics Committee of Harbin Medical University. The male Wistar rats (200‐250 g, derived from the Harbin Medical University's Experimental Animal Center) were induced to myocardial hypertrophy via abdominal aorta coarctation. All animal experiments were conducted in accordance with the protocols approved by the Experimental Animal Ethics Committee of Harbin Medical University. In short, the male Wistar rats were anaesthetized with sodium pentobarbital (40 mg/kg, i.p.), and the abdomen was opened on aseptic conditions. Under sterile conditions, the abdominal aorta was separated for 1 cm segment above the double renal artery, and a diameter of 0.7 mm silver wire was placed on the surface of the abdominal aorta. Then, the abdominal aorta and wire were tightly tied together by surgical silks, and a constriction of the abdominal aorta was provided. The wire was withdrawn from the ligature, so that remaining orifice of the abdominal aorta could close to that of the wire. The rats were divided into three groups randomly: control group, model group and sham group. Sham group underwent laparotomy only, not for abdominal aortic constriction. The ratio of heart weight (HW) to bodyweight (BW) was measured 4 weeks after aortic constriction.

### 2.2 Echocardiographic examination

After 4 weeks of aortic constriction, the rats were anaesthetized with sodium pentobarbital (40 mg/kg, i.p.), for the duration of the transthoracic echocardiographic procedure. All echocardiographic procedures were performed with a Visualsonic Vevo 2100 instrument (2100, VisualSonics, Canada). Left ventricular posterior wall depth (LVPWs), left ventricular anterior wall thickness (LVAWs), left ventricular fractional shortening (LVFS) and left ventricular ejection fraction (LVEF) were calculated. The rats were killed by cervical dislocation, and their hearts were taken away rapidly and washed with ice‐cold 0.9% saline after the echocardiographic measurements. Parts of the hearts were frozen in −80°C refrigerator or fixed in 4% paraformaldehyde 48 hours for later use.

### 2.3 Haematoxylin‐Eosin staining

The left ventricle of male Wistar rat was placed in 4% paraformaldehyde solution at 4°C overnight, then, added 30% sucrose solution. The left ventricle tissue was sunk to the bottom of 30% sucrose solution, embedded in OCT‐freeze medium and frozen at −20°C. Sections of several micron thickness were knifed and transferred to 3‐aminopropyl triethoxysilane‐coated slides. These sections were stained with haematoxylin‐eosin (HE), and the histopathological abnormalities were investigated under the light microscope. The photographs were captured with an Olympus BX60 microscope (Olympus Optical Co. Ltd., Tokyo, Japan).

### 2.4 Cell culture and microRNA transfection

Myocardial cells were isolated and cultured as described.^27^ In brief, the hearts removed from neonatal rats (1to3‐day‐old) were finely minced and placed together in 0.25% trypsin. Collected cell suspensions were centrifuged and resuspended in DMEM supplemented with 10% foetal bovine serum. The resuspension was plated onto culture flasks for 90 minutes, which allowed for preferential attachment of fibroblasts to the bottom of the culture flasks. The cardiomyocytes would be purified through selective adhesion of non‐myocytes at a 90 minutes pre‐plating interval. After 90 minutes, pipette the resuspension into another culture flask for culture at 37°C in a humidified atmosphere containing 5% CO_2_ and 95% O_2_.

Myocardial cells were transfected with the X‐tremeGENE miR‐103 transfection reagent (Roche, Penzberg, Germany) according to the manufacturer's protocol. The target sequence for miR‐103 was 5′‐AGCAGCAUUGUACAGGGCUAUGA‐3′, and the sequence of the miR‐103 inhibitor (AMO‐103) was 5′‐UCAUAGCCCUGUACAAUGCUGCU‐3′. A non‐sense sequence was used as a negative control (N.C) (5′‐UUCUCCGAACGUGUCACGUTT‐3′), and miR‐103 inhibitor negative control (AMO‐N.C) sequence was 5′‐CAGUACUUUUGUGUAGUACAA‐3′. All sequences were synthesized by GenePharma Co. (Shanghai, China). Cells were divided into seven groups: (1) control group; (2) model group: cells were treated with 100 nmol L^−1^ Ang II for 48 hours; (3) miR‐103 group: cells were exposed to 100 nmol L^−1^ Ang II for 48 hours and treated with miR‐103 transfection for 24 hours; (4) AMO‐103 group: cells were exposed to 100 nmol L^−1^ Ang II for 48 hours and treated with miR‐103 and AMO‐103 transfection for 24 hours; (5) N.C group: cells were exposed to 100 nmol L^−1^ Ang II for 48 hours and treated with N.C transfection for 24 hours; (6) AMO‐N.C group: cells were exposed to 100 nmol L^−1^ Ang II for 48 hours and treated with AMO‐N.C transfection for 24 hours; (7) control+miR‐103 group: cells were treated with miR‐103 transfection for 24 hours.

### 2.5 TRPV3 silencing

Cells were plated in 6‐well plates and cultured for 48 hours. Cardiomyocytes were transfected with the X‐tremeGENE small interfering RNA (siRNA) transfection reagent (Roche) according to the manufacturer's protocol. The target sequence for TRPV3 specific siRNA (TRPV3‐siRNA) was 5′‐ACCUGCCUGAUGAAAGCUUTT‐3′. A scrambled siRNA (TRPV3‐sc) sequence was considered as a negative control (5′‐UUCUCCGAACGUGUCACGUTT‐3′). All sequences were synthesized by GenePharma Co. Cells were divided into five groups: (1) control group; (2) model group: cells were treated with 100 nmol L^−1^ Ang II for 48 hours; (3) siTRPV3 group: cells were pre‐treated with TRPV3‐siRNA transfection for 24 hours and finally exposed to 100 nmol L^−1^ Ang II for 48 hours; (4) N.C group: cells were transfected with TRPV3‐sc for 24 hours followed by treatment with 100 nmol L^−1^ Ang II for 48 hours; (5) control+siTRPV3 group: cells were treated with TRPV3‐siRNA transfection for 24 hours.

### 2.6 Western blot

Protein samples were extracted from tissues and cells with the same procedures described previously. Briefly, the heart tissues and myocardial cells were lysed in RIPA buffer and then centrifuged at 4°C 12 000 *g* for 15 minutes. The supernatant was collected, and protein concentrations were determined by bicinchoninic acid (BCA) protein assay (Beyotime, Shanghai, China). Protein extracts were separated by SDS‐PAGE and transferred onto a nitrocellulose membrane. Then, the membranes were blocked and incubated with the following primary antibodies: anti‐BNP antibody (1:1000, Santa Cruz Biotechnology, Santa Cruz, CA, USA), anti‐β‐MHC antibody (1:2000, Sigma, St Louis, MO, USA), anti‐TRPV3 antibody (1:200, Abcam, Cambridge, MA, USA), anti‐Beclin‐1 antibody (1:500, Abcam), anti‐LC3‐II antibody (1:500, Abcam)and anti‐β‐actin (1:2000, Santa Cruz Biotechnology). β‐actin was used as a loading control. After incubation at 4°C overnight, membranes were washed for three times with Tris Buffered Saline with Tween‐20 (TBST), 10 minutes each time, then incubated with secondary antibody for 1 hour at room temperature in the dark. Finally, the membranes were washed for three times with TBST, 10 minutes each time, and scanned by Odyssey Imaging System (LI‐COR Biosciences, Lincoln, NE, USA).

### 2.7 Immunofluorescence staining

Immunofluorescence staining was performed to detect the expression of α‐SMA in myocardial cells. In short, the cells were cultured on coverslips and received the desired treatment. At the end of the treatment, the cells were washed with PBS, fixed with 4% paraformaldehyde and permeabilized using 0.2% Triton^X−100^. Cells were incubated with anti‐α‐SMA at 4°C overnight. Then, the second antibody was incubated in the dark. In the end, DAPI (Beyotime Biotechnology, Shanghai, China) was counterstained for the identification of nucleus. Photographs were acquired using fluorescence microscope (Leica, Heidelberg, Germany).

### 2.8 Real‐time polymerase chain reaction (RT‐PCR)

Total RNA was extracted with Trizol reagent (Invitrogen, Shanghai, China) and reverse transcribed with the one‐step RT‐PCR kit (TransGen Biotech Co., Ltd., Beijing, China) according to the manufacturer's instructions. PCR was performed in a final reaction volume of 25 μL containing 200 ng cDNA, 12.5 μL SYBR Green RT‐PCR Master Mix and 1.25 μL of each of the two primer solutions (10 μmol L^−1^). Parameters for thermal cycling were as follows: 50°C for 2 minutes, 95°C for 15 minutes followed by 40 cycles at 94°C for 15 seconds, 60°C for 30 seconds and 72°C for 30 seconds. Real‐time PCR was performed to determine mRNA expression levels with the ABI PRISM 7500 Sequence Detection System (Applied Biosystems, Foster City, CA, USA) with SYBR Green (TransGen Biotech Co., Ltd.).

### 2.9 Measurement of relative fluorescence intensity of Ca^2+^ signal

Fluorescence measurements in cardiomyocytes have been described previously.^20^ The neonatal rat cardiomyocytes were incubated with a working solution containing 10 μmol L^−1^ Fluo‐3/AM (acetoxymethyl ester form, Molecular Probes, St. Louis, MO, USA) and 0.03% Pluronic F‐127 at 37°C for 40 minutes. Then, the cardiomyocytes were rinsed twice with Tyrode solution to remove the remaining dye. Intracellular calcium concentration was represented by relative fluorescence intensity of Ca^2+^ signal. During the experiment, fluorescent intensity of Fluo‐3/AM in cardiomyocytes was detected for 5 minutes using a laser scanning confocal microscope (Olympus) with excitation at 488 nm and emission at 530 nm. Relative fluorescence intensity was observed in 10 randomly chosen cells to calculate the average fluorescence intensity for all cells.

### 2.10 Tandem mRFP‐GFP fluorescence microscopy

Cells transiently expressing mRFP‐GFP‐LC3 lentivirus were treated as designated and observed by laser microscopy. The number of GFP and mRFP puncta per cell were quantified manually.

### 2.11 Statistical analysis

All data were expressed as mean ± SEM and analysed by using SPSS 19.0 software. Statistical analysis was performed with one‐way ANOVA of variance. If the ANOVA was significant, SNK‐q was used to evaluate the statistical significance of differences between two groups. *P* < 0.05 was considered to be a statistically significant difference.

## 3 RESULTS

### 3.1 Successfully established cardiac hypertrophy models in vivo and in vitro

**Figure 1:**
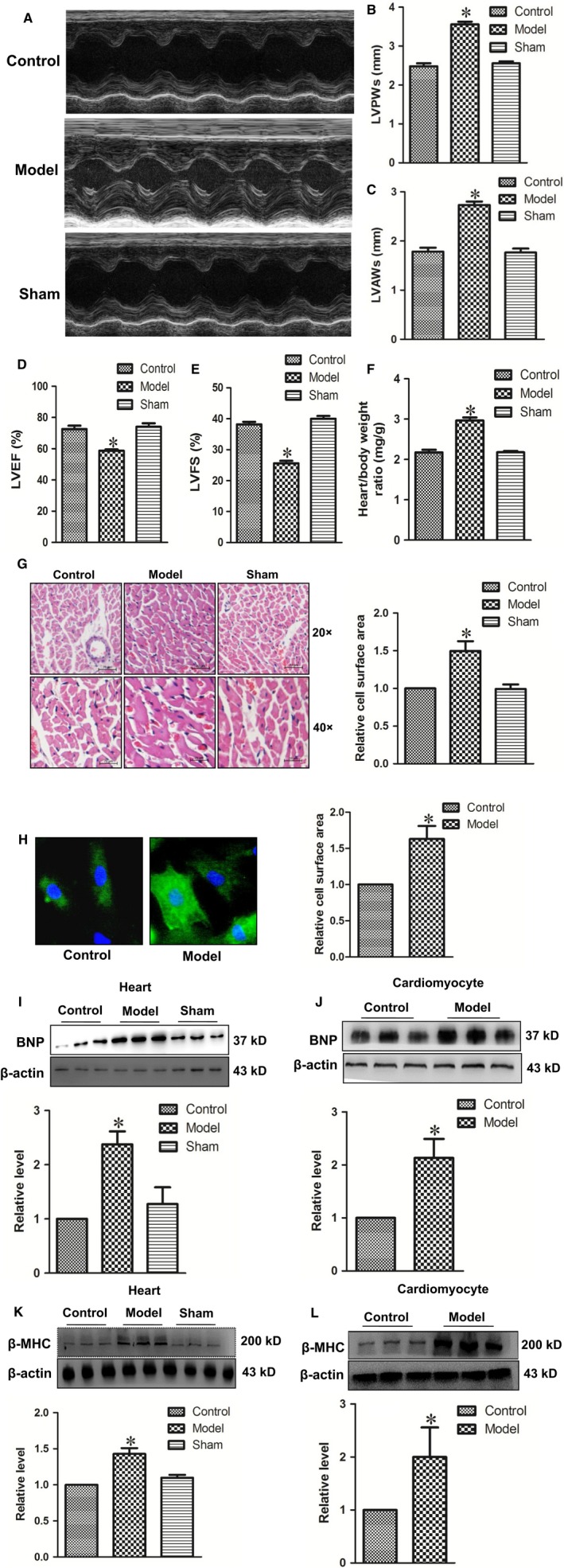
Successfully established cardiac hypertrophy models in vivo and in vitro. The echocardiographic parameters were measured throughout the experiment. (A) Representative M‐mode images of the indicated groups. (B) LVPWs: left ventricular posterior wall depth. (C) LVAWs: left ventricular anterior wall thickness. (D) LVEF: left ventricular ejection fraction. (E) LVFS: left ventricular fractional shortening. (F) Quantitative data of heart‐to‐bodyweight ratio. (G) Histological sections were stained with haematoxylin and eosin (HE) to detect cardiomyocyte hypertrophy (×200 and ×400). (H) Cardiomyocyte surface areas were detected (×200) with α‐SMA antibody (green signal). (I and J) The expressions of BNP protein were measured in vivo and in vitro. (K and L) The expressions of β‐MHC protein were measured in vivo and in vitro. Data were represented by mean ± SEM (n = 3‐6). *P < 0.05 vs control group

To ensure the accuracy of the experiment, firstly we evaluated whether the cardiac hypertrophy models were successfully established in vivo and in vitro. The cardiac hypertrophy models were built by abdominal aorta coarctation in vivo as well as treated with 100 nmol L^−1^ Ang II for 48 hours in vitro respectively. As shown in Figure 1A‐F, there were significant increases in LVPWs, LVAWs and HW/BW, as well as decreases in LVEF and LVFS in the model group, indicating that the cardiac dysfunction was developed in all hypertrophic model rats. Then, cardiomyocyte hypertrophy was assessed by HE and immunofluorescence stain. We found that cell surface areas were both significantly increased in model group in vivo and in vitro (Figure 1G‐H). As we know, BNP and β‐MHC proteins were considered as cardiac hypertrophy signs. Western blot results showed that BNP and β‐MHC protein expressions were significantly increased in hypertrophic hearts and cardiomyocytes (Figure 1I‐L). These results indicated that cardiac hypertrophy models were successfully established in vivo and in vitro.

### 3.2 The activity of autophagy was enhanced in hypertrophic hearts and cardiomyocytes

**Figure 2:**
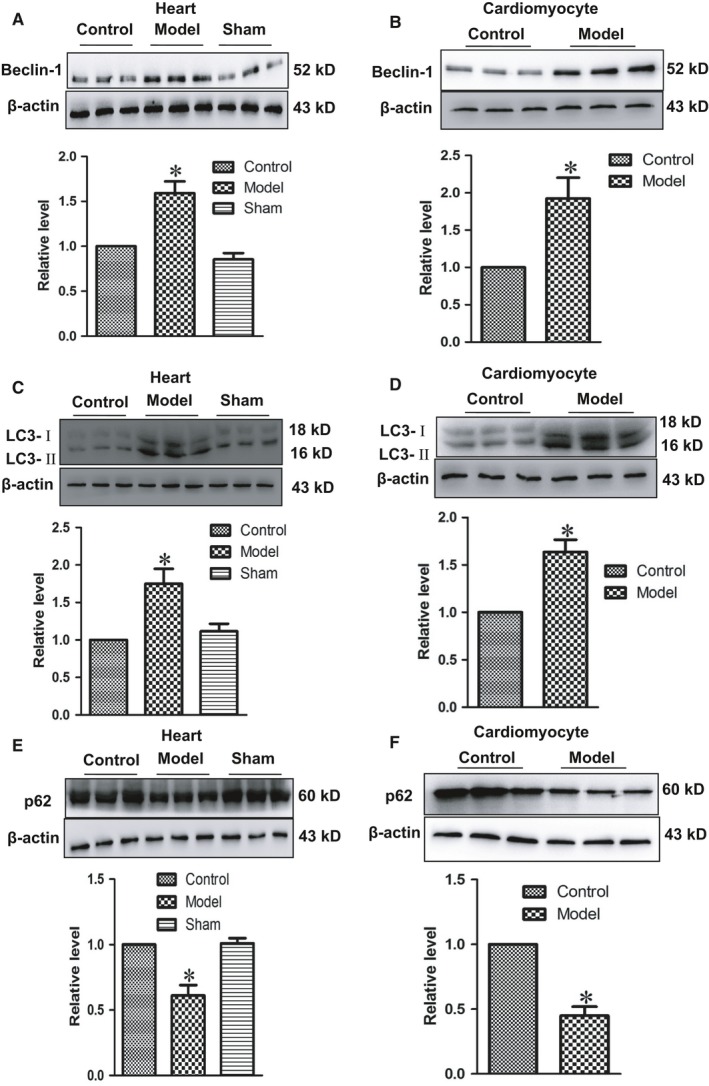
The activity of autophagy was increased in cardiac hypertrophy heart and cultured cardiomyocyte challenged by Ang II 100 nmol L^−1^. (A and B) The expressions of Beclin‐1 protein were measured in vivo and in vitro. (C and D) The expressions of LC3‐II protein were measured in vivo and in vitro. (E and F) The expressions of p62 protein were measured in vivo and in vitro. Data were represented by mean ± SEM (n = 3). *P < 0.05 vs control group

LC3 is recognized as an autophagosome marker. There are two types of LC3 identified: LC3‐I and LC3‐II. LC3‐I is soluble and exists in the cytoplasm, while LC3‐II is a non‐soluble form stably expressed on the double membrane of autophagosomes, which can be biochemically detected to evaluate the level of autophagy. Beclin‐1 also has key roles in autophagy, including regulating the autophagy‐promoting activity of Vps34 and involving in the recruitment of membranes to form autophagosomes. Recently, accumulated evidence indicates that p62 is closely associated with autophagic process. p62 binds to ubiquitinated proteins through ubiquitin‐associated (UBA) domain and delivers them to autophagosomes for degradation. To explore if autophagy contributes to cardiac hypertrophy, levels of autophagic markers Beclin‐1, LC3‐II and p62 were evaluated. In our study, Beclin‐1 (Figure 2A and B) and LC3‐II (Figure 2C and D) proteins were increased remarkably in model group in vivo and in vitro. Meanwhile, p62 expression was dramatically decreased (Figure 2E and F). Above results suggested that autophagy was activated in hypertrophic hearts and cardiomyocytes.

### 3.3 Effects of TRPV3 activation on cardiac hypertrophy

**Figure 3:**
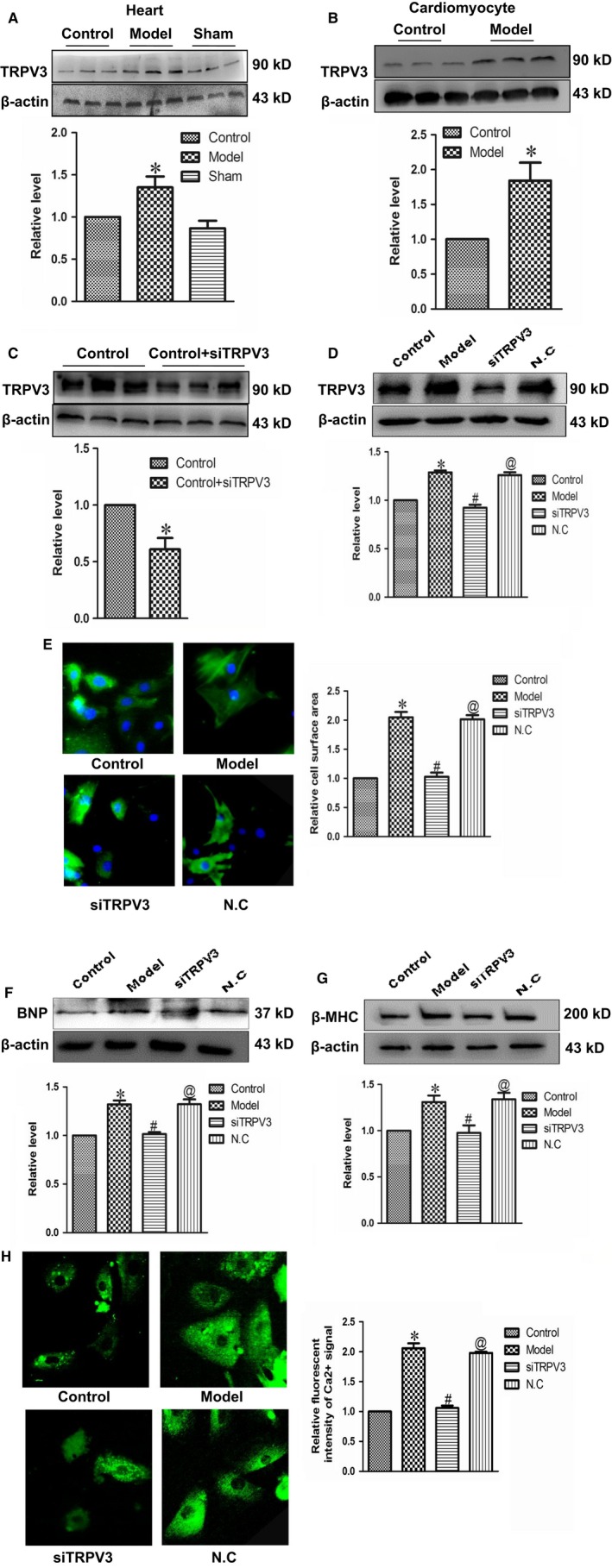
TRPV3 activation promoted cardiac hypertrophy. (A and B) The expressions of TRPV3 protein were measured in vivo and in vitro. (C and D) Successfully silencing TRPV3 by transfecting TRPV3‐siRNA sequence into cultured cardiomyocytes. (E) Silencing TRPV3 reduced the surface area of cardiomyocytes (×200) treated with Ang II. (F and G) Silencing TRPV3 suppressed the expressions of BNP and β‐MHC protein in cardiomyocytes treated with Ang II. (H) Relative fluorescence intensity of Ca^2+^ signal was recorded by laser scanning confocal microscope. Data were represented by mean ± SEM. (n = 3) *P < 0.05 vs control group; ^#^ *P* < 0.05 vs model group; ^@^ *P* < 0.05 vs siTRPV3 group

To better understand TRPV3‐offered detrimental effects on cardiac hypertrophy, cardiac hypertrophy related mark proteins and cell surface areas were monitored. Firstly, we would confirm whether TRPV3 was involved in the regulation of cardiac hypertrophy. The results showed that the protein expressions of TRPV3 in model group were evidently up‐regulated compared with control group in hearts and cardiomyocytes (Figure 3A and B). Secondly, to further evaluate the role of TRPV3 in cardiac hypertrophy, we used siRNA to target TRPV3, and suppressed the expression of TRPV3 successfully (Figure 3C and D). Thirdly, we found that silencing TRPV3 channel significantly decreased cell surface area (Figure 3E) and the expressions of hypertrophy related mark proteins BNP (Figure 3F) and β‐MHC (Figure 3G), as well as inhibited relative fluorescence intensity of Ca^2+^ signal (Figure 3H) compared with model group. Together, these findings indicated that activating TRPV3 channel promoted the occurrence of cardiac hypertrophy.

### 3.4 Effects of TRPV3 activation on the activity of cardiac autophagy

**Figure 4:**
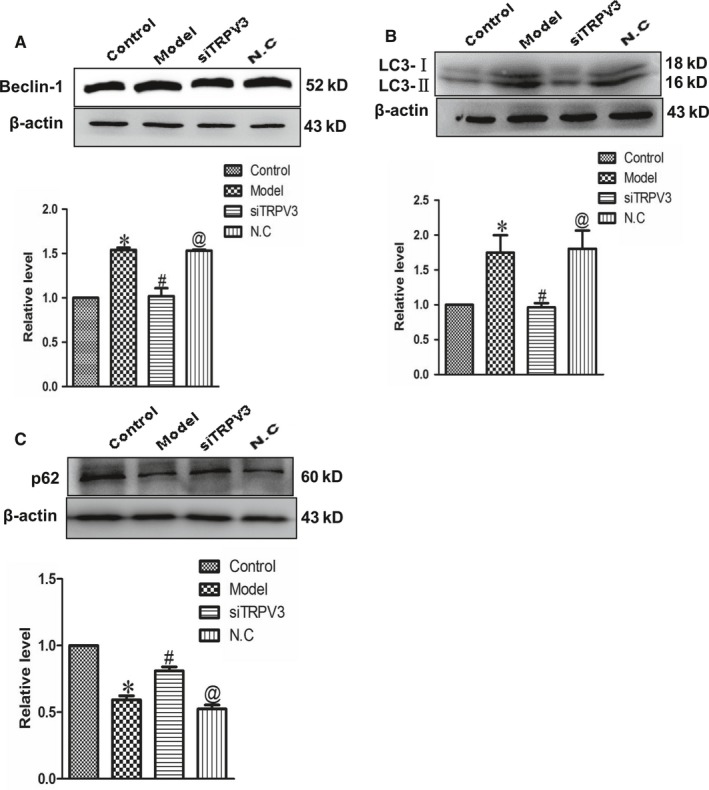
Silencing TRPV3 inhibited cardiac autophagy. (A‐C) The expressions of Beclin‐1, LC3‐II and p62 proteins were measured in cultured cardiomyocytes challenged by Ang II 100 nmol L^−1^. Data were represented by mean ± SEM (n = 3). *P < 0.05 vs control group; ^#^ *P* < 0.05 vs model group; ^@^ *P* < 0.05 vs siTRPV3 group

To further explore the mechanism underneath by which TRPV3 aggravated cardiac hypertrophy, cardiac autophagy was detected. As displayed in Figure 4, autophagy related proteins Beclin‐1 and LC3‐II (Figure 4A and B) were significantly increased, as well as p62 protein (Figure 4C) was decreased in model group, whereas silencing TRPV3 inhibited the expressions of Beclin‐1 and LC3‐II, and enhancing p62 expression. These data indicated that TRPV3 activation might promote cardiac autophagy, finally resulting in cardiac hypertrophy.

### 3.5 Effects of miR‐103 on cardiac hypertrophy

**Figure 5:**
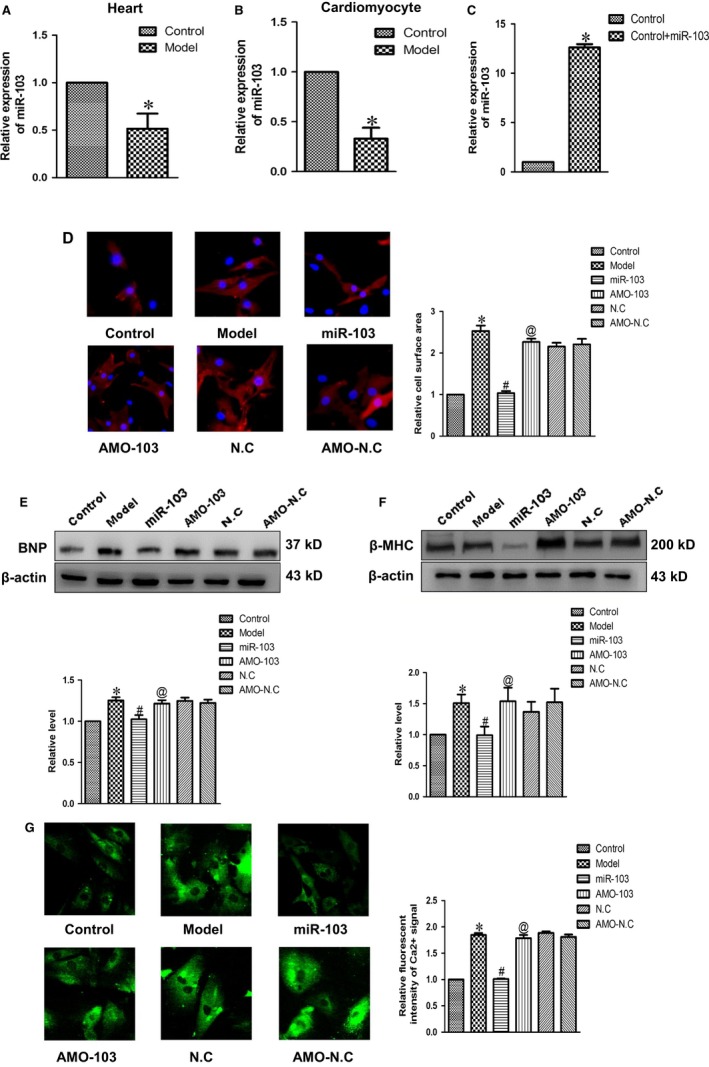
The effect of miR‐103 on cardiac hypertrophy. (A and B) The expressions of miR‐103 in cardiac hypertrophy models in vivo and in vitro were assayed by quantitative real‐time PCR analysis. U6 was used as an internal control. (C) Expression level of miR‐103 in cardiomyocytes transfected with scramble or miR‐103 mimics. (D) Representative photographs of immunofluorescence staining (×200) and statistical histogram of cardiomyocyte area. (E and F) The expressions of BNP and β‐MHC proteins were measured in cultured cardiomyocytes. (G) Forcing overexpression of miR‐103 repressed relative fluorescence intensity of Ca^2+^ signal in cultured cardiomyocytes challenged by Ang II. Data were represented by mean ± SEM (n = 3). *P < 0.05 vs control group; ^#^ *P* < 0.05 vs model group; ^@^ *P* < 0.05 vs miR‐103 group

To determine the effect of miR‐103 on cardiac hypertrophy, we used real‐time PCR to measure the expression of miR‐103 in rat myocardial tissues and neonatal cardiomyocytes. The results were shown in Figure 5A and B, and the expression of miR‐103 in model group was lower than control group obviously in vivo and in vitro respectively. These results suggested that miR‐103 might participate in cardiac hypertrophy regulation. To further investigate the effect of miR‐103 on cardiac hypertrophy, we forced overexpression of miR‐103 in neonatal rat ventricular myocytes (Figure 5C). Overexpressing miR‐103 dramatically inhibited cell surface area (Figure 5D) and the expressions of hypertrophy related mark proteins BNP (Figure 5E) and β‐MHC (Figure 5F), as well as relative fluorescence intensity of Ca^2+^ signal (Figure 5G), compared with model group. These data revealed that miR‐103 had inhibitory effect on cardiac hypertrophy.

### 3.6 Effects of miR‐103 on the activity of cardiac autophagy

**Figure 6:**
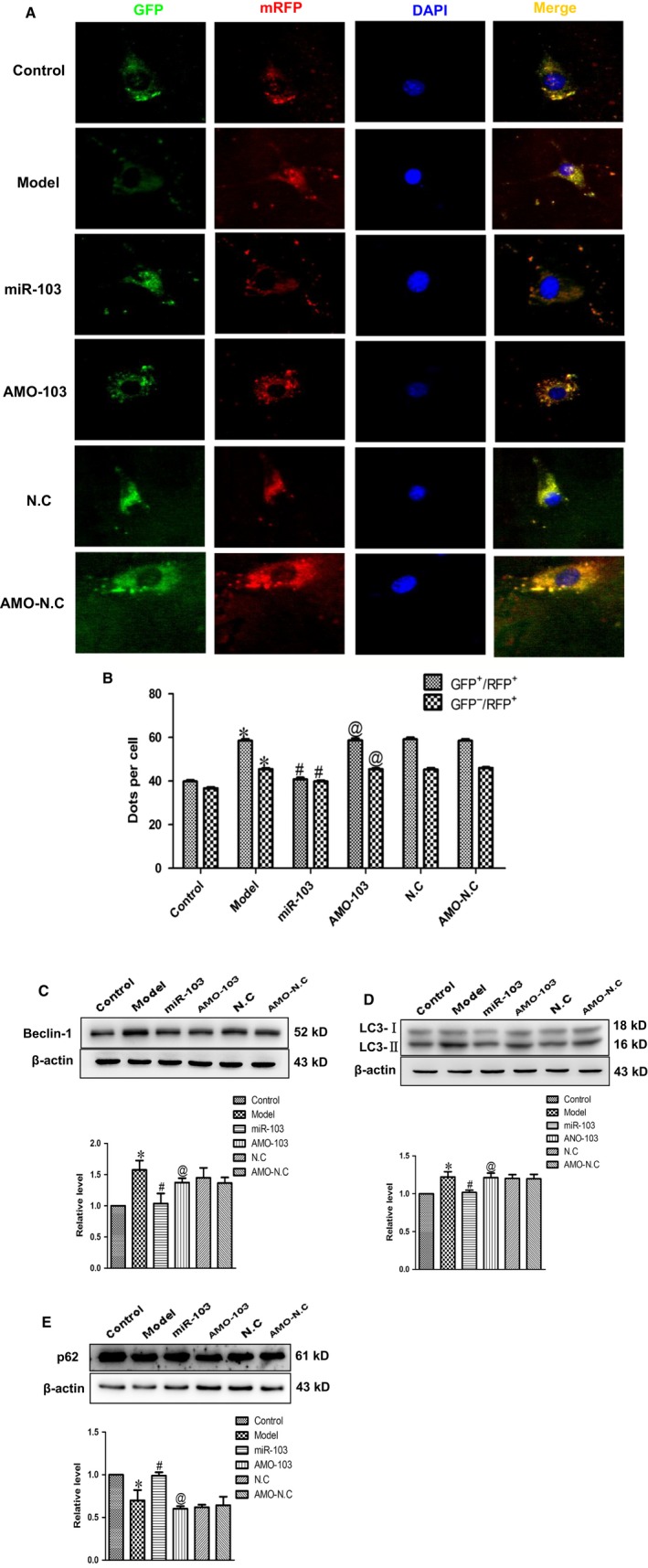
The effect of miR‐103 on cardiac autophagy. Forcing overexpression of miR‐103 inhibits autophagic flux. (A) Representative images of mRFP‐GFP‐LC3 puncta (×200). (B) The numbers of GFP ^+^/RFP ^+^ (yellow) and GFP ^−^/RFP ^+^ (red) dots per cell were quantified. (C‐E) Forcing overexpression of miR‐103 restrained Beclin‐1 and LC3‐II, and promoted p62 protein expressions. Data were represented by mean ± SEM (n = 3). *P < 0.05 vs control group; ^#^ *P* < 0.05 vs model group; ^@^ *P* < 0.05 vs miR‐103 group

To further examine the potential mechanism of miR‐103 overexpression protection against cardiac hypertrophy, cardiac autophagy was examined in cardiomyocytes. Autophagy is a dynamic process that involves autophagosome formation and lysosome degradation. To assess the effect of miR‐103 on autophagic flux, the mRFP‐GFP‐LC3 assay was employed. In merged images, the yellow and red puncta represented autophagosomes and autolysosomes, respectively, because mRFP but not GFP retained fluorescence in the acidic environment of lysosomes. When autophagic flux was blocked in the autophagosome–lysosome fusion stage, only GFP^+^/RFP^+^ (merged as yellow) puncta accumulate. In our study, miR‐103 overexpression resulted in decreases of both yellow and red puncta, whereas knockdown of miR‐103 induced the accumulation of both yellow and red puncta (Figures 6A and B). Consistent with autophagic flux results, autophagy related proteins Beclin‐1 (Figures 6C) and LC3‐II (Figures 6D) were significantly elevated and the levels of p62 were decreased (Figures 6E) in model group. However, overexpression of miR‐103 significantly ameliorated the activity of cardiac autophagy. These results implied that miR‐103 suppressed cardiac hypertrophy partly via reducing the activity of cardiac autophagy.

### 3.7 TRPV3 gene is a target of miR‐103

**Figure 7:**
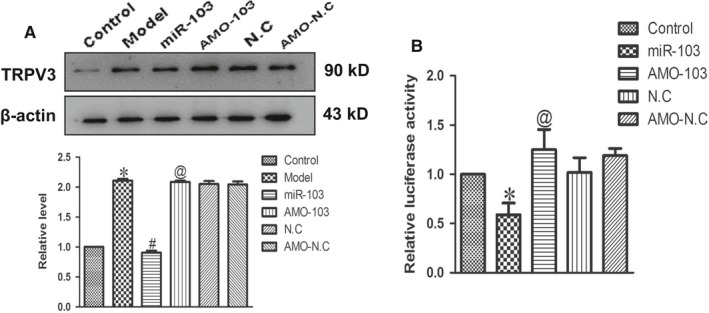
TRPV3 was the direct target of miR‐103 in cardiac hypertrophy. (A) Effect of miR‐103 on the protein expression of TRPV3 in cultured neonatal rat cardiomyocytes. (B) Luciferase reporter activities of chimerical vectors carrying luciferase gene and a fragment of TRPV3 3′‐untranslated region from rat containing the binding sites of miR‐103. Data were represented by mean ± SEM (n = 3‐5). *P < 0.05 vs control group; ^#^ *P* < 0.05 vs model group; ^@^ *P* < 0.05 vs miR‐103 group

Subsequently, we need to examine whether miR‐103 directly targeted TRPV3. We used targetscan software to predict, and found a conserved binding site for the miR‐103 in the 3′ UTR of the TRPV3 gene. To further test this binding profile, miR‐103 was transfected into cultured neonatal rat ventricular myocytes, and the protein level of TRPV3 induced by Ang II was remarkably reduced. Conversely, TRPV3 was significantly up‐regulated when AMO‐103 was transfected into neonatal rat ventricular myocytes, which indicates that TRPV3 was negatively regulated by miR‐103 (Figure 7A). To further verify that the miR‐103 directly targeted TRPV3, we prepared luciferase constructs carrying the TRPV3 3′ UTR. Co‐transfection of the miR‐103 with the luciferase reporter vector into HEK293 cells caused a sharp decrease in luciferase activity compared with transfection of the luciferase vector alone. The miR‐103‐induced depression of luciferase activity was rescued by an AMO‐103 used to delete miR‐103 (Figure 7B). These results experimentally revealed that TRPV3 was a direct target of miR‐103.

## 4 DISCUSSION

Cardiac hypertrophy is an adaptive process to cellular stress that involves changes in both gene expression and sarcomeric organization. It is characterized by the increases in the volume and diameter of myocardial cells. Initially, this compensation can improve cardiac function, but long‐term pathological changes can predispose individuals to heart failure, arrhythmia and sudden cardiac death. Despite, it is believed to be mediated by signalling molecules which transduce the stress signals from the environment into different cellular compartments, the underlying molecular mechanisms that couple hypertrophic signals initiated at the cell membrane to the reprogramming of cardiomyocyte gene expression remain poorly understood. The main findings of the current study are that miR‐103 exerts the inhibitory effect on cardiac hypertrophy partly via reducing cardiac autophagy by directly targeting TRPV3.

Interest in autophagy has increased in recent years as this highly conserved cellular process had been proved to implicate in a growing number of diseases including infection, cancer, neurodegeneration and ageing.^28^ At the same time, it has also been reported that autophagy can promote the activation of inflammatory factors,^29^ and plays a role in neurodegenerative diseases such as Parkinson's disease.^30^ In recent years, studies have shown that autophagy is involved in the development of a variety of cardiovascular diseases. Huang reported that berberine alleviated cardiac ischaemia/reperfusion injury by inhibiting excessive autophagy in cardiomyocytes.^31^ In addition, it has been reported that excessive cardiac autophagy can lead to cardiac hypertrophy.^32^ Consistent with previous reports, our results indicated that excessive cardiac autophagy exacerbated cardiac hypertrophy in vivo and in vitro models.

The activation of TRPV3 channel is involved in the development of hyperphagia and obesity in obesity‐prone rats by reducing food intake,^33^ and also plays an important role in scars with post‐burn pruritus by thymic stromal lymphopoietin (TSLP).^34^ In addition to the above‐mentioned effects, the TRPV3 channel is also involved in the regulation of cardiovascular diseases. Our previous studies have found that activation of TRPV3 channels can aggravate cardiac fibrosis in the pressure‐overloaded rat hearts.^20^ The present study further indicated that the expression of TRPV3 protein was significantly enhanced, both in vivo and in vitro of rat cardiac hypertrophy models. To further confirm the regulation of TRPV3 on cardiac hypertrophy, we used siRNA technology to silence TRPV3. After knockdown of TRPV3, the myocardial cell area was significantly reduced compared with the model group. At the same time, the expressions of cardiac hypertrophy marker proteins BNP and β‐MHC, as well as intracellular calcium signal, were also significantly inhibited. Recently, accumulate evidence indicates that autophagy is activated in aortic banding model.^35^, ^36^ Intriguingly, our results demonstrated that the expressions of Beclin‐1 and LC3‐II were also decreased, and p62 was increased, the important regulatory proteins of autophagy after TRPV3 knockdown. As we all know, intracellular calcium is an important regulator of autophagy. Therefore, we speculate that the activation of TRPV3 channels can increase the intracellular calcium concentration, and thus promote myocardial cell autophagy, ultimately leading to cardiac hypertrophy.

MicroRNAs are endogenous regulators of gene expression. Considering that cardiomyocyte hypertrophy, a key cellular event in hypertrophic heart, depends on gene expression. And it is reasonable to suggest that miRNAs may be involved in pressure overload‐induced cardiac hypertrophy. It should be noted that several studies have demonstrated that miRNAs protect against cardiac hypertrophy. MiR‐133a was down‐regulated in thyroid hormone‐regulated cardiac hypertrophy,^37^ and miR‐23a acted downstream of NFATc3 to regulate cardiac hypertrophy,^38^ and overexpression of miR‐27b could induce cardiac hypertrophy.^39^ Wang reported that miR‐103/107 could regulate programmed necrosis and myocardial ischaemia/reperfusion injury through the FADD pathway.^40^ However, whether miR‐103 plays a role in cardiac hypertrophy has not been addressed. The results of this experiment showed that miR‐103 expression was decreased when cardiac hypertrophy occurred. This finding suggested that miR‐103 was involved in the occurrence of cardiac hypertrophy. To further investigate the role of miR‐103 in cardiac hypertrophy, we used both gain‐ and loss‐of‐function techniques on miR‐103 expression to explore the regulatory effect of the miR‐103 on antihypertrophic action. Our results indicated that forcing overexpression of miR‐103 significantly reduced cardiomyocyte cell area and intracellular calcium signal. Simultaneously, forcing overexpression of miR‐103 in neonatal rat ventricular myocytes dramatically inhibited mark proteins BNP and β‐MHC (cardiac hypertrophy), and Beclin‐1 and LC3‐II expressions, as well as autophagic flux activation (cardiac autophagy). In contrast, AMO‐103 significantly enhanced their expressions and activation in neonatal rat ventricular myocytes. These findings prompted that miR‐103 reduced intracellular calcium, thereby suppressed cardiac autophagy, finally inhibited cardiac hypertrophy. Furthermore, by using targetscan software, the current study predicted a putative miR‐103 binding sequence in the TRPV3 mRNA. To validate that TRPV3 was a target of miR‐103, we transfected miR‐103 or inhibitors into neonatal rat ventricular myocytes, and found that miR‐103 could affect TRPV3 protein level. Subsequently, it was verified that TRPV3 was a direct miR‐103 target using luciferase reporter assays in HEK293 cells. These results indicated that miR‐103 directly modulated TRPV3 expression by binding to the 3′ UTR of TRPV3 and, thus, confirmed that TRPV3 was a novel target of miR‐103.

**Figure 8:**
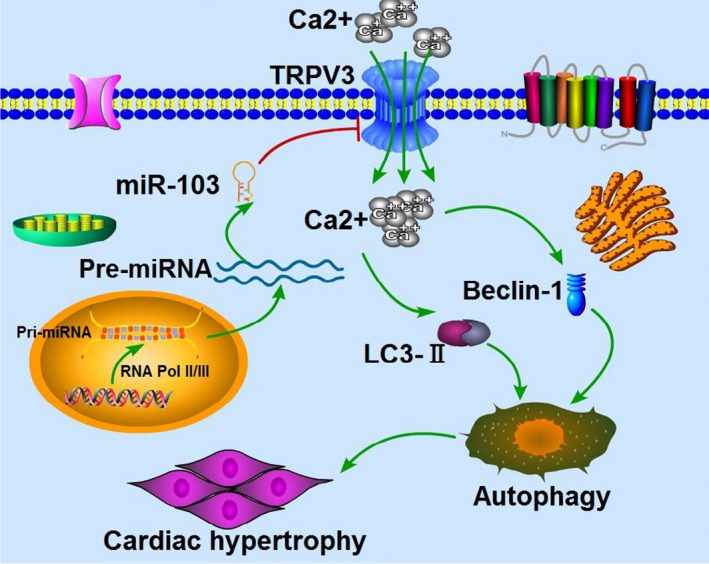
Schematic diagram for the proposed miR‐103‐anti‐hypertrophic signalling pathways

In summary, miR‐103 could suppress the expression of TRPV3 by binding to the 3′ UTR, thereby reducing relative fluorescence intensity of Ca^2+^ signal, leading to the decrease in the activation of cardiac autophagy and ultimately inhibiting the development of cardiac hypertrophy (schematized in Figure 8). Our studies revealed a novel hypertrophic signalling pathway (miR‐103/TRPV3/autophagy), and manipulation of this signalling axis may provide innovative approaches to prevent cardiac hypertrophy.

## CONFLICT OF INTEREST

The authors declare that they have no conflict of interest.
